# Factors related to functional exercise capacity amongst people with HIV in Durban, South Africa

**DOI:** 10.4102/hsag.v26i0.1532

**Published:** 2021-04-29

**Authors:** Penelope M. Orton, Dudu G. Sokhela, Kathleen M. Nokes, Joseph D. Perazzo, Allison R. Webel

**Affiliations:** 1 Department of International Education and Partnerships, Durban University of Technology, Durban, South Africa; 2 Department of Nursing, Faculty of Health Sciences, Durban University of Technology, Durban, South Africa; 3 Department of Nursing, City University of New York (CUNY) Graduate Center, New York, United States of America; 4 Department of Nursing, University of Cincinnati, Cincinnati, United States of America; 5 Frances Payne Bolton School of Nursing, Faculty of Health Sciences, Case Western Reserve University, United States of America

**Keywords:** South Africa, KwaZulu-Natal, eThekwini Metropolitan, HIV, physical activity, walking

## Abstract

**Background:**

People with HIV (PWH), who engage in regular physical activity, have improved fitness, muscular strength, body composition, health-related quality of life and mental health symptoms, but PWH have amongst the lowest physical activity levels of those with any chronic health condition. Furthermore, there is scant evidence examining these relationships in PWH in Africa.

**Aim:**

To address these critical gaps, this cross-sectional descriptive research study examined the relationships between demographic, HIV-related, anthropometric factors, neighbourhood walkability and physical activity, amongst PWH in Durban, South Africa.

**Setting:**

Respondents (*N* = 100) were receiving primary healthcare in six eThekwini nurse-run municipal clinics.

**Methods:**

Self-reported socio-demographic data were collected, and HIV-related medical data were extracted from respondent’s medical charts. Height and weight were measured to calculate the body mass index (BMI, kg/m^2^); neighbourhood walkability was measured on the Neighbourhood Environment scale; and physical activity, specifically functional exercise capacity, was measured by the 6-min walk test (6MWT).

**Results:**

On average, respondents were black African, female, approximately 38 years old and unemployed; men were of normal weight whilst women were overweight. Only 65% of the respondents reached the age- and sex-predicted distance during the 6MWT. Correlational analyses did not reveal any significant relationships between the functional exercise capacity and socio-demographic, HIV-related factors or anthropometric measures.

**Conclusion:**

South African PWH do not reach their predicated walking distance on the 6MWT. Engaging community agencies to promote walking as both a means of transportation and leisure physical activity may decrease the risks of a sedentary lifestyle and improve progression towards recommended physical activity targets.

## Introduction

Despite the well-known benefits of regular and intense physical activity, people with HIV (PWH) have the lowest levels of physical activity. According to the recent World Health Organization (WHO) guidelines on physical activity and sedentary behaviour (WHO 2020), physical activity can occur during multiple domains, including leisure time, occupation, education, household activities and transportation. The transportation domain refers to physical activity, such as walking, which is performed for the purpose of getting to and from places. In South Africa, almost one in five adults walk entirely to their destination, and approximately a third walk to receive medical services (Statistics South Africa 2014). Walking can be a particularly important form of transportation, and exercise, in environments with unreliable public transportation and limited economic resources.

Physical activity is any bodily movement produced by skeletal muscles resulting in energy expenditure. Everyone performs physical activity to sustain life, but the amount of physical activity one engages in is subject to personal choice and may vary considerably from person to person, as well as for a given individual, over time (Caspersen, Powell & Christenson 1985). Functional exercises are those that are embedded into everyday tasks and improve lower body strength, balance and motor performance (WHO 2020). Functional capacity reflects the ability to perform activities of daily living, such as walking, which requires sustained aerobic activity. Functional capacity, exercise capacity and exercise tolerance are often used interchangeably in the literature (Arena et al. 2007).

According to the Statistics South Africa (2020), approximately 13% of the South African population is living with HIV/AIDS. Treatments for HIV can result in a decline in muscle function and reduced physical activity (Oliveira et al. 2020). This reduced physical activity may be especially pronounced in women who are at the centre of the economic production for the family in most African communities (Vancampfort, Stubbs & Mugisha 2018). Physical activity has been associated with improvements in cardiorespiratory fitness (maximal oxygen consumption, exercise tolerance), muscular strength, body composition, health-related quality of life, reduced symptoms of depression and anxiety, and no change in HIV viral load or cluster of differentiation 4 (CD4)+ T-cell count in PWH (WHO 2020). Physical activity can help overcome the negative effects of multiple chronic comorbidities and antiretroviral therapy, but PWH often do not reach levels recommended by the physical activity guidelines (Voigt, Cho & Schnall 2018).

There is also an emerging literature on the additional beneficial effects of physical activity amongst those with HIV. Dufour et al. (2018) examined the relationship between physical activity and neurocognitive function, and found that both HIV-infected and HIV-uninfected persons, who consistently engaged in physical activity, maintained better neurocognitive function over time compared with those who participated inconsistently or who did not report any physical activity. Forde et al. (2018) explored the incidence of metabolic syndrome between a matched sample of HIV-infected and HIV-uninfected men in Dublin, Ireland. Antiretroviral therapy can cause metabolic syndrome, leading them to examine if there was a relationship between objectively measured physical activity and metabolic syndrome, and they found that, although HIV-infected respondents were more physically active than the HIV-negative control group, the metabolic profile between groups was similar, suggesting that physical activity provided a protective effect against metabolic syndrome. Kiama et al. (2018) reported similar findings in an urban population of PWH in Nairobi, Kenya.

A meta-analysis of the effectiveness of aerobic exercise in PWH found that it is safe and leads to significant improvements in cardiorespiratory fitness (O’Brien et al. 2016). Lopes et al. (2019), in exploring how exercise affects the vascular system of PWH, found beneficial effects on vascular function, reactivity and redoxin in active respondents of a similar age, which provides additional support for physical activity. A review of physical activity levels of PWH living in Sub-Saharan Africa (Vancampfort et al. 2018) found that lower physical activity was consistently associated with older age, lower number of CD4 T-cells/µL, higher HIV load and higher body mass index (BMI, kg/m^2^). Whilst there are mixed findings about the association of gender and physical activity, duration of HIV antiretroviral therapy use was consistently unrelated to the physical activity level.

The 6-min walk test (6MWT) has been used to measure functional exercise capacity in a number of studies. Casanova et al. (2011) conducted a cross-sectional, international, multicentre study with normal volunteers aged 40–49 years and found that the average man walked 650 m and the average woman walked 600 m. In one older sample of PWH (Oursler et al. 2018), the average baseline distance walked during the 6MWT was 551 m (standard deviation [SD] = 33 m). In a different HIV-infected sample with an average age of 52 years, men walked an average distance of 415 m whilst women walked an average distance of 367 m (Oliveira et al. 2018). Webel et al. (2019) used the 6MWT to measure cardiorespiratory fitness in a community-living sample with HIV/AIDS and found that respondents from the United States and Thailand (*N* = 702) walked an average of 402 (± 104) m with expected differences by sex. Men walked an average of 422 m whilst women walked an average of 369 m, a significant difference (< 0.001). However, both men and women achieved similar percentages (68% vs. 69%; *p* = 0.96) of their sex- and age-predicted distance on the 6MWT.

There is limited data using an objective measure of functional exercise capacity in an HIV-infected sample in South Africa. An international study of physical activity in PWH (Webel et al. 2019) was conducted by the International Nursing Network for HIV Research (Holzemer 2007) in a variety of sites in the United States and Thailand. The authors participated in the international study; this article presents data drawn from HIV-infected persons living in Durban, South Africa.

## Methods

### Study design

This cross-sectional correlational study explored the relationships between demographic, HIV-related, anthropometric factors, accessibility of business and facilities, and functional exercise capacity of community-living PWH living in Durban, South Africa.

### Setting

Respondents were recruited at six eThekwini municipal primary care clinics in Durban, South Africa. These primary healthcare (PHC) clinics are nurse-led and services are offered free of charge by the Provincial Department of Health (60%) and 40% by the local government authority, also known as the municipality. The eThekwini district is situated in KwaZulu-Natal (KZN), one of South Africa’s nine provinces and consists of a diverse society which faces various social, economic and health challenges (eThekwini Municipality 2011). The total population of eThekwini district is 3 442 361 and makes up a third of the population of KZN; black people constitute 73.8% of this population (City Population n.d.). eThekwini district is an industrialised area with a high number of informal settlements and high disease burden. According to the South African National HIV Prevalence, Incidence, Behaviour and Communication Survey, KZN has the highest prevalence of HIV in South Africa (18.1%) (Simbayi et al. 2019).

### Study population and sampling strategy

A convenience sampling was used to recruit community-living HIV-infected persons receiving primary care in municipal health centres serving large numbers of PWH (*N* = 100). Eligible respondents were ≥ 18 years of age, had a HIV-positive laboratory test, no medical contraindications for exercise, walked without assistive devices, and understood English.

### Data collection

#### Measures

**Demographic, human immunodeficiency virus-related, and anthropometric characteristics:** All respondents completed self-reported socio-demographic items (age, gender, race); medical chart abstraction identified the number of years that a respondent had been living with HIV, current CD4 T-cell count and current HIV viral load. Respondents’ height and weight were measured by the study personnel and used to calculate the BMI. BMI measures height and weight, and is a simple, inexpensive and non-invasive test. Anthropometric measures of waist–hip ratio were taken following procedures outlined by the WHO (2011). Three measures of both waist and hip circumference were taken and used to calculate waist–hip ratio (waist measure divided by hip measure). The average of the three calculated ratios was used to develop the final waist–hip ratio used in our analysis. The waist–hip ratio has been used as a predictor of cardiovascular disease and diabetes (WHO 2011).

**Accessibility of business and facilities:** Respondents completed the Neighbourhood Environment Walkability Scale (NEWS) (Cerin et al. 2006) which was designed to measure residents’ perceptions of the environmental attributes of their local area. One subscale assesses proximity to stores and facilities and includes questions about types of businesses or facilities in their neighbourhood. Respondents were asked ‘about how long would it take them to get from your home to the nearest business or facility listed below if they walked to them?’ on a Likert-type scale with six options that ranged from 1 min to 5 min to more than 30 min or do not know. Respondents reported on the number of minutes required to walk to a supermarket, fruit/vegetable market, pharmacy, laundromat and bus stop.

*Functional exercise capacity* was assessed by the widely used and validated self-paced 6MWT that provides valuable information in clinical practice and can be assessed when resources are limited (Ross et al. 2016). Standardised instructions and encouragement (Singh et al. 2014) are commonly given and respondents are asked to walk as far as possible in 6 min. The primary outcome was walking distance which was recorded in metres.

The research team included two fluent English and Zulu speakers and all the members of the team were trained as registered professional nurses. One of the research team members met with eligible respondents and reviewed an informed consent document that described the study’s purpose, procedures, risk and potential benefits. Respondents gave permission to access their medical record for HIV-related data. After confirming understanding, the staff obtained written informed consent and were available to assist respondents to complete the study instruments. Study staff collected anthropometric assessments of height and weight (for BMI) and waist–hip ratio and the 6MWT to measure walking capacity. Upon completion of the procedures, respondents received a thank you letter and R100.00, as approved by the institutional research ethics committee and only given on completion of all procedures. Respondents were not advised that they would receive a thank you letter and small gift before giving written informed consent. All data collection occurred between September and November 2018.

### Data analysis

Collected data were entered into a central REDCap database created for the international study data, which was housed at the coordinating site, where it was cleaned and regularly checked for quality. REDCap is a secure web application for building and managing online surveys and databases that are specifically geared to support data capture for research studies and operations (Harris et al. 2019).

Data were cleaned and statistical assumptions were tested using frequency distributions and univariate statistics. The authors used means, standard deviations and median values to determine central tendency. We ran Spearman’s rho correlations to correct for non-normally distributed/categorical variables and Pearson Product Moment correlations for continuous measures. Viral load and CD4 T-cell counts were measured in cells/mm^3^ and undetectable viral loads were coded at ‘0’. The authors used dummy coding (e.g. ‘0’ and ‘1’) for categorical demographic variables, including race, gender and employment type. Pearson’s chi-square analyses were done to determine whether there were significant differences between men and women on demographic variables. All data were analysed at a significance level of *p ≤* 0.05 using Stata^®^ version 15 (StataCorp 2017). REDcap’s calculation feature was used to ensure accurate conversion of respondents’ weight and height and 6MWT distances (metres).

### Ethical considerations

Institutional Ethics approval was received by the Durban University of Technology (REC 87/16) and permission was granted by the eThekwini Municipality Department of Health to collect data at a selection of their primary health clinics.

## Results

### Characteristics of the sample

The average respondents were black African, female, approximately 38 years old and unemployed (see Table 1). Significant differences were observed in average BMI for men (24.21 kg/m^2^), which indicates normal weight, and 27.84 kg/m^2^ for women indicating overweight (National Heart, Lung and Blood Institute n.d.) (Table 1). Eighty per cent of the respondents (*n* = 79) were taking HIV antiretroviral therapy and 80% had an undetectable HIV viral load; the average CD4 T-cell count was 505 per cubic millimetre of blood, indicating that the immune system was functioning relatively effectively. There were significant differences between women and men on HIV-related factors. Women had been diagnosed with HIV significantly longer, had significantly higher CD4/T cells, but no difference in viral load was observed (Table 1).

**TABLE 1:** Sample characteristics (*N* = 100).

Characteristics	Overall (*N* = 100)	Men (*n* = 18)	Women (*n* = 82)	*p* (difference)
Age (mean)	38.46	44.67	37	0.063
SD	10.45	12.82	9.33	-
Range (in years)	21–77	29–77	21–62	-
Race/ethnicity	-	-	-	-
Black African (%)	99	100	99	-
Mixed race	1	-	1	-
Employment status				
Working now (%)	33	44	31	0.254
Looking for work, unemployed (%)	52	50	51	0.446
Student (%)	6	-	7	-
Keeping house (%)	5	-	6	-
Disabled (%)	2	-	2	-
Retired (%)	2	6	1	0.234
No permanent housing (%)	42	56	43	0.741
Height (cm)	166	168	165	0.073
Weight (kg)	69.55	62.8	71	0.000*
BMI (kg/m^2^)	27.22	24.21	27.84	0.000*
Years diagnosed with HIV	-	-	-	< 0.001
Mean	5.50	3.67	5.85	-
SD	4.61	3.79	4.70	-
Years (range)	1997–2018	1997–2018	2002–2018	-
Most recent CD4/T-cell count	-	-	-	< 0.001
Mean	511	366	544	-
SD	312.07	217.05	322.27	-
HIV viral load	-	-	-	0.198
Undetectable (%) (50 c/mL)	70	61	72	-
Detectable (%) (50 c/mL)	18	22	17	-
6-min walk test walked (m)	416.24	436.38	411.82	0.454
SD	100.88	129.29	93.91	-
6-min walk test of goal walked (%)	65.30	64.90	65.38	0.943
SD	15.62	22.00	13.97	-

BMI, body mass index; SD, standard deviation; HIV, human immunodeficiency virus; CD4, cluster of differentiation 4.

* , *p* ≤ 0.05.

In characterising their neighbourhood walkability, 59% of the respondents could walk to a supermarket within 30 min; 54% to a fruit/vegetable market; 44% to a pharmacy; 43% to a laundromat and 91% could walk to a bus stop which, considering the transportation choices in the community, was probably a shared taxi (see Table 2). Sixty-five per cent of the sample reached the set distance during the 6MWT. The average total distance walked was 416 m which, considering the predicted distance was 643 m, is 65% of the desired number of metres walked over the 6 min. Women walked an average of 411 m compared with men who walked 436 m (*t* = 0.935, degree of freedom (*df*) = 98, *p* = 0.352).

**TABLE 2:** Neighbourhood environment scale (*N* = 100)†.

Type of business or facility	1 min – 5 min	6 min – 10 min	11 min – 20 min	20 min – 30 min	30+ min or don’t know
Supermarket (%)	06	11	17	25	41
Fruit/vegetable market (%)	09	10	14	21	46
Pharmacy (%)	04	05	14	21	56
Laundromat (%)	05	06	08	24	57
Bus stop (%)	42	30	11	08	08

† , Numbers rounded off so might not add up to 100%.

The correlational analyses showed no significant relationships between any of the study variables (gender, age, BMI, -waist–hip ratio, CD4 T-cell count or viral load) and the distance walked (see Table 3). We entered all variables into a multiple linear regression model (Table 4) using our functional exercise capacity measure as a constant. The relationships between the possible predictors and functional exercise capacity remained non-significant [*F*[6, 70] = 0.91, *p* = 0.91, *R*^2^ = 0.0723).

**TABLE 3:** Correlations between predicted distance on 6-min walk test and selected demographic, anthropometric and HIV-related variables.

Characteristics	Gender	Age	Waist–hip ratio	BMI (kg/m^2^)	CD4	Viral load
6MWT % of predicted distance	*r* = 0.068	*r* = 0.167	*r* = 0.044	*r* = 0.140	*r* = 0.190	*r* = 0.104
	*p* = 0.519	*p* = 0.112	*p* = 0.681	*p* = 0.183	*p* = 0.085	*p* = 0.354

6MWT, 6-minute walk test; BMI, body mass index; CD4, cluster of differentiation 4.

**TABLE 4:** Multiple linear regression model.

Characteristics	*B*†	*β*‡	SE§	*t*	*p*¶
Functional exercise capacity	34.47	-	17.93	1.92	0.059
Gender	0.102	0.003	5.49	0.02	0.985
Age	0.215	0.141	0.194	1.11	0.271
Waist–hip ratio	16.10	0.103	18.22	0.88	0.380
BMI	0.149	0.066	0.276	0.54	0.591
CD4 count	0.009	0.176	0.006	1.48	0.145
Viral load	0.000	0.029	0.000	0.24	0.812

BMI, body mass index; SE, standard error; CD4, cluster of differentiation 4.

† , Unstandardised beta coefficient;

‡ , Standardized beta coefficient;

§ , Standard error;

¶ , Significance measured at the level of *p*≤ 0.05.

## Discussion

The purpose of this research was to assess whether demographic and anthropometric factors were related to functional exercise capacity in a community-living sample of PWH; no significant relationships were found. Mabweazara et al. (2019) studied physical activity in a similar HIV-infected sample living in Cape Town, South Africa, using the Global Physical Activity Questionnaire (GPAQ), and found that gender, educational level and employment status were associated with self-reported physical activity. Prior research had found that the most important factor influencing the 6MWT in healthy subjects was older age (Oliveira et al. 2018), but there was no relationship between age and the 6MWT in this sample, perhaps because this South African sample was younger with an average age of 38 years. Female gender has been associated with reduced physical activity (Oliveira et al. 2018; Webel et al. 2019) but not in this sample where there was no significant difference between men and women and distance walked.

The BMI of our female respondents was significantly greater than that of our male respondents (Table 2), which is consistent with other research. Body weight directly affects the work/energy required to perform the 6MWT (Singh et al. 2014) but, in this sample, BMI was not related to results on the 6MWT. HIV-related factors such as immune function as measured by CD4-T cells approached statistical significance (*p* = 0.085; Table 3) in that those with lower CD4-T cells, which indicates more immune damage, had less walking capacity which is consistent with the findings of Vancampfort et al. (2018). The earliest efforts to identify HIV infection in South Africa were aimed at women of child-bearing years (South African Department of Health 2003), and it is not surprising that women were diagnosed significantly longer than men. Yet, the observed difference in immune function, with higher CD4/T cell count, needs further exploration because there was no difference in viral load between women and men (Table 1). Perhaps the expected relationships between age, gender, BMI, and HIV-related factors and functional exercise capacity were not found because the sample was relatively homogeneous on those variables. Unlike other findings (Webel et al. 2019), 65% of both the men and the women respondents reached the desired distance. Walking as a mode to increase physical fitness is particularly important in this setting because the majority of respondents reported being able to walk to essential services within 30 min.

In 2013, the United Nations set three targets which became known as 90-90-90 (UNAIDS 2014). Specifically, the targets were that by 2020, 90% of all PWH would know their HIV status, receive sustained antiretroviral therapy and have viral suppression (UNAIDS 2014). South Africa is making significant progress towards those targets. According to the South African National HIV Prevalence, Incidence, Behaviour and Communication Survey, 2017, 85% of respondents living with HIV knew their status, 71% were on treatment and 86% were virally suppressed (Simbayi et al. 2019:154). The sample in this research reflects the success of this target because the average CD4-T cell count was relatively normal and viral suppression was achieved for 80% of the sample in which a viral load test was documented on their medical record. This community-living sample of PWH walked both as a means of transportation and leisure activity. Sixty-five per cent reached the desired distance during the 6MWT, but the actual distance walked was further than in the international US and Thai samples (Webel et al. 2019).

### Limitations

Socio-demographic data were self-reported, and the sample was relatively homogeneous with respect to race, gender and age range but reflected the HIV-infected population in Durban. Convenient, cross-sectional rather than random sampling was used but data were collected from six municipal health clinics providing HIV-related care in the area.

## Conclusion

Physical activity has a significant role, in many cases comparable or superior to pharmacological interventions, in the prevention and management of more than 40 conditions such as diabetes mellitus, cancer, cardiovascular disease, obesity, depression, Alzheimer disease and arthritis (Lobelo et al. 2018). It is estimated that the prevalence of HIV disease in South Africa may be as high as 19% (World Bank 2021). With the advent of access to HIV treatment, large numbers of the population are aging, and HIV will be just another comorbidity (Kasaie et al. 2021). Physical activity can prevent conditions such as metabolic syndrome which is common in PWH who are taking antiretroviral medications.

According to the WHO guidelines on physical activity, adults living with HIV should limit their sedentary time and aim to engage in more than the recommended levels of moderate-to-vigorous intensity physical activity than other adults (WHO 2020:58). Fitness data in South Africa are limited, and, similar to the results found in a younger population living in Soweto (Prioreschi et al. 2017), BMI was significantly higher in a female compared with male HIV-infected sample. As the 6MWT walking distance of this community-living population was only 65% of the desired goal, interventions to increase physical fitness are indicated. Routine engagement in health behaviours, including physical activity, helps to prevent and manage comorbid health conditions and syndromes of aging common amongst PWH (Montoya et al. 2019). Any physical activity is better than none. Adults who sit less and do any amount of moderate-to-vigorous physical activity gain some health benefits although, for significant health benefits, more strenuous activity is necessary. As many South Africans walk as a means of transportation, additional in-depth research is indicated to explore strategies on how walking can be increased. Many smartphones have free apps that measure steps walked and, whilst this might not be the most reliable method of measuring steps, it could be a start because many South Africans have smartphones (Gilbert 2019).

Involvement of community agencies such as church, women’s, or youth groups, sports clubs and burial societies (Campbell, Williams & Gilgen 2002; Matuku & Kaseke 2014) can be leveraged to encourage increased physical activity amongst PWH. Healthcare providers could identify the local voluntary associations in the areas served by the particular health clinic and encourage these groups to develop low-cost physical activity opportunities. For example, in the eThekwini municipal area there are many parks and open spaces which have outdoor gymnasiums, paths and areas where sports such as soccer and netball can be played. Black South Africans have a culture of music and dance which could be used as a strategy to attract PWH to increase physical activity by offering traditional music and dance as a group activity in the open park spaces. Assistance could be provided to encourage the use of local facilities, develop leadership in the association to lead the activities and provide a conduit to refer PWH into these physical activities.

As highlighted in the WHO guidelines (2020), there is limited evidence on physical activity from low- and middle-income countries, economically disadvantaged or underserved communities, and in people living with disability and/or chronic disease. This current research contributes to filling that gap by using an objective functional exercise capacity assessment measure that identified that physical activity levels need improvement in PWH living in Durban, South Africa.
